# Elevated alpha1-acid glycoprotein in gastric cancer patients inhibits the anticancer effects of paclitaxel, effects restored by co-administration of erythromycin

**DOI:** 10.1007/s10238-015-0387-9

**Published:** 2015-09-10

**Authors:** Yoshinao Ohbatake, Sachio Fushida, Tomoya Tsukada, Jun Kinoshita, Katsunobu Oyama, Hironori Hayashi, Tomoharu Miyashita, Hidehiro Tajima, Hiroyuki Takamura, Itasu Ninomiya, Masakazu Yashiro, Kousei Hirakawa, Tetsuo Ohta

**Affiliations:** 1 Department of Gastroenterological Surgery, Kanazawa University Graduate School of Medical Science, 13-1 Takara-machi, Kanazawa, 920-8641 Japan; 2 Department of Surgical Oncology, Graduate School of Medicine, Osaka City University, 1-4-3 Asahi-machi, Abeno, Osaka, 545-8585 Japan

**Keywords:** α1-Acid glycoprotein, Gastric cancer, Peritoneal carcinomatosis, Paclitaxel, Erythromycin

## Abstract

Paclitaxel (PTX) which easily elutes into ascites is widely used to treat gastric cancer patients with peritoneal carcinomatosis (PC), but clinical outcomes are suboptimal. Increased concentrations of α1-acid glycoprotein (AGP), an important drug-binding protein, have been reported in the plasma and ascites of cancer patients. This study sought to clarify whether AGP binds to PTX and alters its anticancer effects. AGP concentrations were measured in the serum and ascites of gastric cancer patients with PC and in the serum of healthy volunteers. The in vitro effects of AGP and AGP plus erythromycin (EM) on PTX were evaluated by MTT assays in the gastric cancer cell lines. We also measured AGP concentrations in the ascites of PC model mice and examined the effects of EM plus PTX on PC. The mean AGP concentrations in the serum and ascites of gastric cancer patients with PC were 1524 and 834 μg/mL, respectively, higher than the mean AGP concentration of 650 μg/mL observed in the sera of healthy volunteers. AGP > 400 μg/mL significantly suppressed the cell growth inhibitory effect of PTX in vitro, but the co-administration of EM restored it. Elevated AGP concentrations were observed in the ascites of PC model mice. Administration of PTX alone did not markedly diminish PC, whereas co-administration of PTX and EM significantly reduced PC (*p* = 0.011). AGP is an important regulatory factor modulating the anticancer activity of intraperitoneal PTX. The co-administration of PTX and EM may be effective in treating gastric cancer patients with PC.

## Background

Gastric cancer is one of the most common malignant diseases worldwide [1]. Although mortality rates have declined, advanced gastric cancer remains life-threatening [2]. Metastasis of gastric cancer frequently includes peritoneal carcinomatosis (PC), and gastric cancer patients with PC have a dismal prognosis [3].

In Japan, the standard first-line treatment for patients with advanced gastric cancer consists of S-1, an oral dihydropyrimidine derivative of 5-fluorouracil (5-FU), plus cisplatin [4]. Gastric cancers, however, are only moderately sensitive, and PC is relatively resistant, to systemic chemotherapy. Although few clinical studies have assessed survival in gastric cancer patients with PC, the median survival time (MST) of these patients treated with sequential methotrexate and 5-FU therapy was reported to be 5 months [5]. Paclitaxel (PTX) is often used to treat gastric cancer patients with PC because it can disseminate easily, with a high rate of passage from the systemic circulation into the peritoneal cavity [6]. Intraperitoneal PTX (IP PTX) has been reported successful in several patients [7, 8], prompting the ongoing phase III PHOENIX-GC trial, assessing the effectiveness of IP PTX in gastric cancer patients with PC. So it has not been recognized as a standard chemotherapy, and intravenous PTX is a standard now. The randomized phase II JCOG0407 (Japan Clinical Oncology Group) trial was conducted to test the effects of weekly intravenous PTX as second-line treatment in which gastric cancer patients with PC, reported an MST of 7.7 months [9]. To date, therefore, intravenous PTX has not shown satisfactory clinical outcomes in gastric cancer patients with PC.

The abundant presence of fibrous components in PC results in severe complications, including ileus, hydronephrosis, and obstructive jaundice. This is why treating gastric cancer patients with PC is difficult. Thus, the management of fibrosis is also crucial in these patients. Transforming growth factor-β (TGF-β) has a pivotal function in the progression of tissue fibrosis. Because low-dose PTX can significantly suppress TGF-β signaling and decrease stromal fibrosis [10], PTX should be a key drug in the treatment of gastric cancer patients with PC, making it necessary to increase the antitumor effects of PTX.

Drugs in the peripheral circulation are present as free compounds or bound reversibly to plasma proteins, polysaccharides, and lipids. In general, only the unbound fraction has pharmacodynamic activity because only free, unbound drug in plasma can diffuse across biologic barriers and be transported to their sites of action [11, 12]. A large fraction of PTX binds to serum proteins, including albumin and α1-acid glycoprotein (AGP). In fact, binding data for PTX to AGP were calculated in previous report [13]. AGP, also called orosomucoid, is an acute-phase protein, and changes in its concentration have been found to influence the free plasma concentration of drugs without affecting their total plasma concentrations. Thus, AGP concentration alters the distribution and metabolism of drugs [14]. Serum concentrations of AGP have been found to increase several fold in response to local inflammatory stimuli, and AGP concentrations were observed to increase in both the plasma and ascites of cancer patients [15, 16]. These findings suggested that AGP concentrations are likely elevated in gastric cancer patients, reducing the unbound fraction of PTX. AGP concentrations were reportedly increased in patients with idiopathic pulmonary fibrosis, and increased AGP was found to suppress the activity of the anti-fibrotic agent imatinib. However, erythromycin (EM) can compete with imatinib in binding AGP, abrogating the AGP-mediated inhibition of imatinib [17].

We therefore examined whether the levels of AGP were higher in gastric cancer patients with PC than in normal volunteers and whether AGP inhibited the activity of PTX in vitro. We also assessed whether EM competed with PTX to bind AGP in vitro and in vivo. AGP may be a cause of drug resistance to PTX, and co-administration of PTX and EM, which competes its drug resistance, may be a new encouraging treatment for gastric cancer patients with PC.

## Methods

### Reagents

PTX was purchased from Bristol-Myers Squibb Company (Tokyo, Japan). EM and AGP were purchased from Sigma-Aldrich Co. (Tokyo, Japan).

### Patients

Serum samples were obtained from 20 gastric cancer patients with PC and from 20 healthy volunteers. Ascites samples were also obtained from 20 gastric cancer patients with PC. This research was performed in accordance with the Declaration of Helsinki. Written informed consent for use of clinical samples and healthy volunteer’s samples, as required by the Institutional Review Board at Kanazawa University, Japan, was obtained from all enrolled patients and volunteers.

### Cell lines and cell culture

OCUM-2MD3, a cell line derived from a human scirrhous gastric cancer, with high peritoneal-seeding activity, was kindly provided by the Department of Surgical Oncology of Osaka City University of Medicine. NUGC-3, a cell line derived from human poorly differentiated gastric cancer, was purchased from National Institute of Biomedical Innovation (Osaka, Japan). Cells were seeded in 75-cm^2^ dishes (Becton–Dickinson, Tokyo, Japan). OCUM-2MD3 cells were cultured in 10-mL Dulbecco’s modified Eagle’s medium (DMEM, Life Technologies, Tokyo, Japan) supplemented with 10 % heat-inactivated fetal bovine serum (FBS) (Nichirei Bioscience Inc., Tokyo, Japan), 100 IU/mL penicillin, 100 mg/mL streptomycin (Life Technologies Tokyo, Japan), at 37 °C in a humidified atmosphere of 5 % CO_2_ in air. The culture medium for NUGC-3 cells was Roswell Park Memorial Institute (RPMI) 1640 medium (Life Technologies) with 15 % FBS, and the other additives were same as above.

### Cell growth assay

OCUM-2MD3 and NUGC-3 cell viabilities in response to PTX, AGP, and/or EM were determined by standard 3-(4,5-dimethylthiazol-2-yl)-2,5-diphenyltetrazolium bromide (MTT) assays. Cells were seeded at 5 × 10^3^ per well in 96-well plates and incubated for 24 h at 37 °C in a humidified environment containing 5 % CO_2_. These cells were subsequently incubated with PTX, AGP, and EM where indicated, for 48 h; the supernatant was removed; and 100 μL of culture medium and 20 μL of MTT solution (CellTiter 96 Aqueous One Solution Cell Proliferation Assay; Promega, Tokyo, Japan) were added to each well. These cells were incubated for 3 h, and the absorbance at 490 nm was analyzed using a microplate reader (Bio-Rad 550; Bio-Rad, Tokyo, Japan). Each sample was assayed in triplicate in each experiment.

### Animals and xenograft model

Male 6-week-old BALB/c nu/nu mice were purchased from Charles River Laboratories Inc. (Yokohama, Japan) housed under specific pathogen-free conditions and fed standard chow pellets and water ad libitum. All experiments adhered to the Standard Guidelines for Animal Experiments at Kanazawa University.

To introduce xenografts into these mice, OCUM-2MD3 cells were harvested from subconfluent cultures, collected by centrifugation, and re-suspended in serum-free DMEM at 1 × 10^7^ cells/mL, with 1 × 10^7^ cells injected into the peritoneal cavity of each mouse.

### AGP analysis

AGP concentrations in human serum and ascites were determined by nephelometry (SRL Institute, Tokyo, Japan). AGP concentrations in the ascites of nine xenografted mice were examined every 3 days by single radial immunodiffusion assays (Ecos Institute, Ohsaki, Japan).

### Co-administration of PTX and EM to mice xenograft

To evaluate whether EM reactivates the antitumor effects of PTX in vivo, OCUM-2MD3 cells were injected into the peritoneal cavity of mice on day zero. Five mice were administered saline, five received intravenous PTX (5 mg/kg each on days 7 and 14), and five received intravenous PTX and subcutaneous EM (5 mg/kg each on days 7–17). All mice were killed on day 17, and the number and weight of the metastatic nodules were determined.

### Statistical analysis

Values are expressed as mean ± standard deviation (SD). Data sets were compared using Student’s *t* test or one-way analysis of variance with Tukey’s post hoc comparison. Statistical analyses were performed using SPSS statistics 19 software (IBM, Tokyo, Japan), with *p* values less than 0.05 considered statistically significant.

## Results

### Elevated AGP concentrations in serum and ascites of gastric cancer patients with PC

**Fig. 1:**
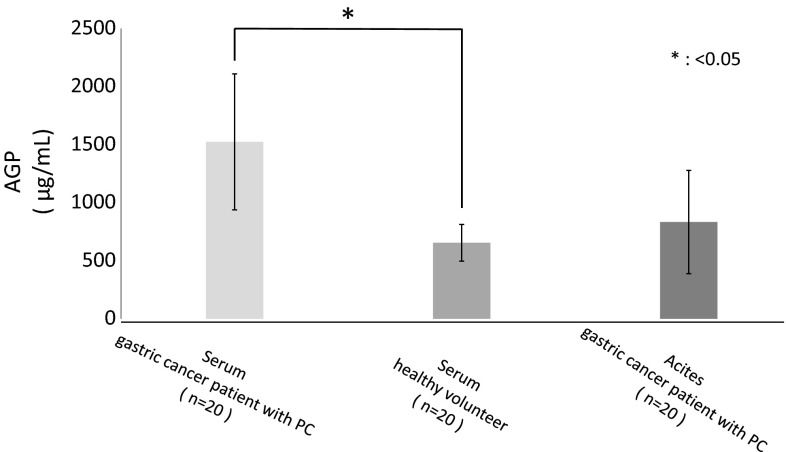
Concentrations of AGP in the serum of gastric cancer patients with PC were about 2.5-fold higher than those of healthy volunteers. The mean concentration of AGP was higher in ascites of gastric cancer patients with PC than in the serum of healthy volunteer, but this difference was not statistically significant. Data are presented as mean ± SD. *p < 0.05 versus serum of healthy volunteers

A comparison of serum AGP concentrations in cancer patients with PC and healthy volunteers found that AGP concentrations were about 2.5-fold higher in the formers than in the latters (1524 ± 586 vs. 650 ± 158 μg/mL, *p* = 0.0049) (Fig. 1). Although the mean concentration of AGP was higher in ascites of gastric cancer patients with PC than in the serum of healthy volunteer, this difference was not statistically significant.

### Elevated AGP concentration in the ascites of a PC model

**Fig. 2:**
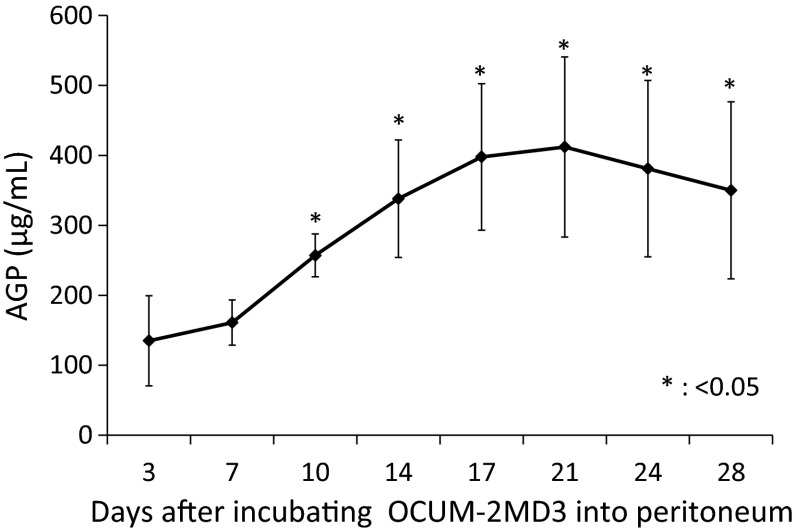
AGP concentrations in ascites of our xenograft model mouse increased with cancer progression, starting on day 10 and reaching a maximum of 400 μg/mL on day 21. Data are presented as mean ± SD. *p < 0.05 versus day 3

To determine whether AGP level changed during cancer progression, AGP concentrations were measured in the ascites fluid of mice intraperitoneally injected with OCUM-2MD3 (1 × 10^7^) cells. AGP concentrations increased with cancer progression, starting on day 10 and reaching a maximum of 400 μg/mL on day 21 (Fig. 2).

### AGP inhibits the activity of paclitaxel in vitro

**Fig. 3:**
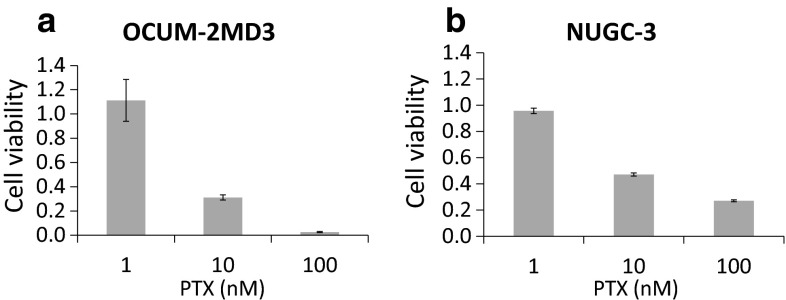
Effect of PTX was assessed by MTT assays. **a** OCUM-2MD3 cell viability, **b** NUGC-3 cell viabilities. Results reported as mean ± SD of three independent experiments

**Fig. 4:**
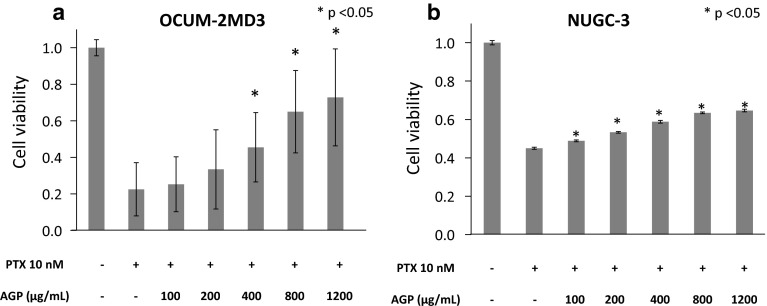
Effect of AGP (0–1200 μg/mL) on PTX-suppressed conditions was assessed by MTT assays. **a** OCUM-2MD3 cell viability, **b** NUGC-3 cell viability. Results reported as mean ± SD of three independent experiments. *p < 0.05 versus cells added culture medium alone

Using MTT assays, we found that 100 and 10 nM PTX inhibited the growth of OCUM-2MD3 cells and NUGC-3 cells in vitro, whereas 1 nM PTX did not adequately (Fig. 3a, b). We subsequently examined whether 0–1200 μg/mL AGP inhibited the in vitro activity of 10 nM PTX, a concentration equivalent to that in ascites during treatment of patients with intravenous PTX [6]. The addition of AGP suppressed the cell growth inhibitory effect of PTX in a dose-dependent manner, with AGP concentrations higher than 400 μg/mL showing statistically significant effects in both OCUM-2MD3 cells and NUGC-3 cells (Fig. 4a, b).

### EM competes with PTX for AGP binding in vitro

**Fig. 5:**
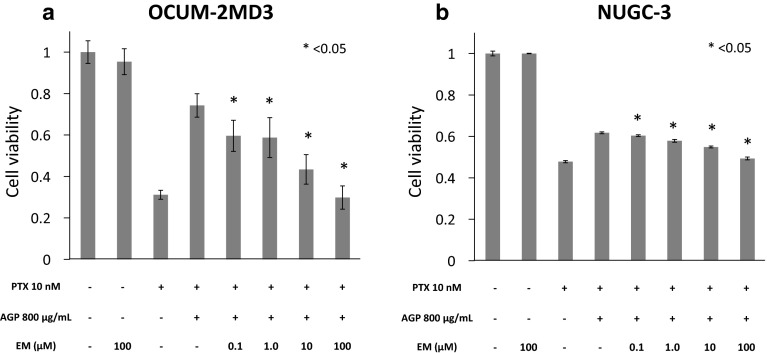
Effect of the co-administration of PTX and EM on OCUM-2MD3 and NUGC-3 cell viabilities in a high-AGP environment was determined using MTT assays. OCUM-2MD3 and NUGC-3 cells were incubated with EM (0.1, 1, 10, 100 μM), AGP (800 μg/mL), and PTX (10 nM), and cell viabilities were measured. Data are presented as mean ± SD of three independent experiments. *p < 0.05 versus cells treated with PTX and AGP

**Fig. 6:**
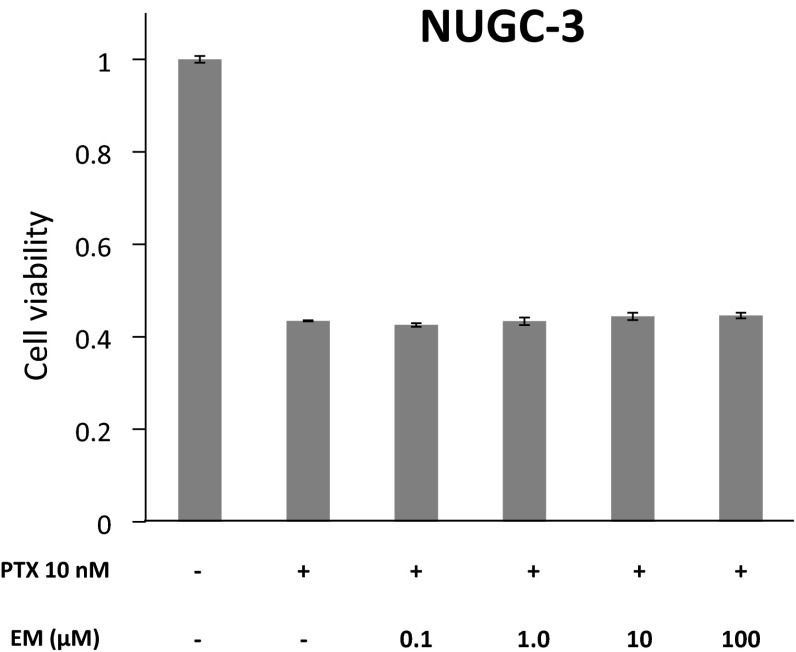
Effect of co-administration of PTX and EM without AGP on NUGC-3 cell viability was determined using MTT assays. NUGC-3 cells were incubated with EM (0.1, 1, 10, 100 μM), and PTX (10 nM), and cell viabilities were measured. The difference of cell viability between the group treated with PTX alone and the groups treated with PTX plus EM was not statistically significant. Data are presented as mean ± SD of three independent experiments

To examine the effect of EM on AGP-mediated suppression of PTX’s activity in vitro, the viabilities of OCUM-2MD3 cells and NUGC-3 cells cultured with EM (0.1, 1, 10, and 100 μM), AGP (800 μg/mL), and PTX (10 nM) were determined using MTT assays. We also examined whether EM without AGP affected the cell growth inhibitory effect of PTX. The addition of EM in a high-AGP environment significantly reactivated the cell growth inhibitory effect of PTX in a dose-dependent manner (Fig. 5a, b). In contrast, EM without AGP did not affect the cell growth inhibitory effect of PTX (Fig. 6).

### Co-administration of EM enhances the antitumor effects of PTX in a mouse PC model

**Fig. 7:**
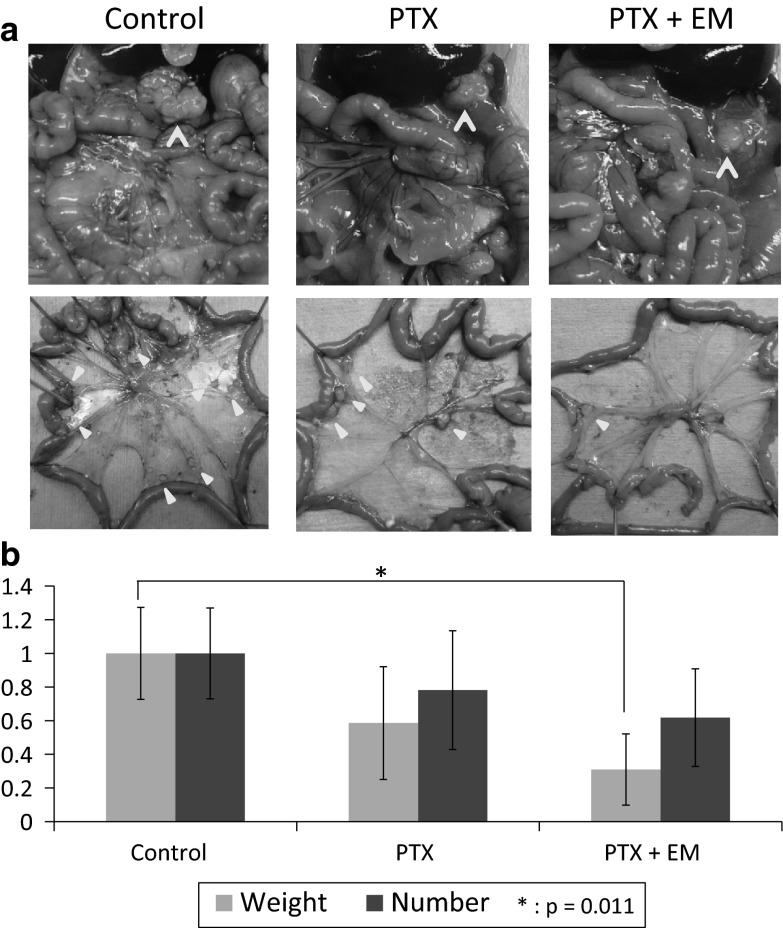
Xenograft model was made by incubating OCUM-2MD3 into nu/nu mice intraperitoneally. Effect of PTX plus EM on metastatic nodules was assessed. **a** Macroscopic views of peritoneal nodules (*arrow head*). **b** Relative number and weight of metastatic peritoneal nodules in the xenograft model. Data are shown as mean ± SD. *p < 0.05 versus untreated group

PTX alone or PTX plus EM tended to reduce the number of peritoneal nodules in mice, but the differences were not statistically significant. In contrast, PTX plus EM, but not PTX alone, significantly reduced the weight of metastatic nodules (Fig. 7a, b).

## Discussion

This study showed that AGP concentrations in the ascites fluid of a mouse model of gastric cancer with PC increased with cancer progression and that elevated AGP suppressed the cell growth inhibitory effects of PTX. We also demonstrated that co-administration of EM, which competed with PTX to bind AGP, restored the cell growth inhibitory effects of PTX in vitro and in vivo.

Many drugs bind reversibly to serum proteins, with bound and unbound fractions being in equilibrium. AGP is an important drug-binding protein, with a molecular weight of around 43 kDa [18]. AGP is negatively charged at physiological pH, and binds mostly basic drugs. AGP is thought to contain three subunits at its drug-binding site; these subunits were not completely separated but significantly overlapped and influenced one another, enabling AGP to bind acidic, basic, and neutral drugs [19–22].

The physiological role of AGP is still not completely understood. Immunosuppressive protein (IAP), a type of AGP, has also been found in the serum and ascites of cancer patients, and IAP has been reported to suppress several immune responses including phytohemagglutinin-induced lymphocyte blast formation and mixed lymphocyte reactions [23]. Elevated IAP may trigger the induction of suppressive macrophages [24]. Moreover, serum IAP concentration in gastric cancer patients has been reported to increase with advancing cancer stage, suggesting that IAP may be useful in estimating the immunological status of these patients [25]. Elevated AGP may have a similar function, inducing immune tolerance in gastric cancer patients.

PTX is widely used to treat gastric cancer patients, especially those with PC, but its efficacy is suboptimal. PTX metabolism is catalyzed mainly by CYP3A4 and 2C8 [26, 27], and it is substrate for ATP-binding cassette transporter such as P-glycoprotein and multidrug resistance-associated protein (MRP) [28, 29]. The competition for these enzymes or overexpression of these transporters causes drug resistance to PTX. EM undergoes extensive hepatic metabolism and is metabolized by CYP3A4 and substrate for P-glycoprotein and MRP affecting metabolism of another drugs [30]. In our study, changing the concentration of EM without AGP did not affect the cell growth inhibitory effect of PTX, while co-cultured with AGP reactivated PTX in a dose-dependent manner. This means EM did not potentiate PTX by the way of CYP3A4, P-glycoprotein, or MRP, but the way of competing with PTX for binding AGP. In brief, increased AGP is associated with the drug resistance to PTX. This is consistent with previous reports that AGP inhibited the activity of molecularly targeted drug and another anticancer drug [17, 31, 32]. The binding of drug to serum proteins is an important determinant of the pharmacokinetics. For drugs that circulate mostly in a bound state, even a small change in the extent of protein binding can have a large effect on drug activity. AGP may have great influence on PTX, because about 90 % of this drug binds to plasma proteins [33]. The mean AGP concentration in the ascites of gastric cancer patients was 834 μg/mL, which was higher than in the serum of healthy volunteers. Moreover, AGP concentration in mouse ascites increased with the growth of the peritoneal nodules. These results suggest that AGP concentrations in the ascites of gastric cancer patients increase with tumor progression, a finding consistent with result showing that AGP concentration in a mouse model of leukemia increased in proportion to tumor load [31].

We found that AGP > 400 μg/mL significantly inhibited the activity of 10 nM PTX in vitro. Because the mean AGP concentration in the ascites of gastric cancer patients with PC was 834 μg/mL, the activity of intravenously administered PTX may be suppressed by elevated AGP in the peritoneal cavity. PTX concentrations in the ascites of advanced gastric cancer patients, who were administered 80 mg/m^2^ of PTX intravenously, were reported to be 35 nM after 12 h and 14 nM after 72 h [6]. In vitro, we found that 10 nM of PTX was inhibited by AGP, indicating that the antitumor effect of intravenous PTX in the peritoneal cavity of gastric cancer patients would be suppressed by elevated AGP.

The addition of another agent to the primary antitumor drug may increase the antitumor effect or decrease the side effects of the latter. This process, called biochemical modulation, is thought to be due to the second agent, called the “modulator,” altering the pharmacokinetics and/or metabolism of the antitumor agent. For example, 5-FU or methotrexate has been administered together with leucovorin. To reactivate the antitumor effect of PTX that was suppressed by AGP, we added EM as a modulator. Fourteen-membered macrolides, including EM, have been reported to compete with molecularly targeted drugs in binding AGP and restoring their activity [16, 30]. We found that EM concentrations >0.1 μM significantly reactivated the cell growth inhibitory effect of PTX (10 nM) suppressed by AGP (800 μg/mL). These concentrations of EM are equivalent to the serum concentrations resulting from the administration of standard dosages to mice and humans [34–36]. Finally, we demonstrated that co-administration of PTX and EM was more effective than PTX alone in the PC mouse model. The negative effect of AGP and the positive one of EM are predicted to be applied not only to the cancer cells but also to the normal cells. However, they have probably little effect on normal cells because PTX originally provides antitumor effect inhibiting active cell division of cancer cells rather than normal cells. Actually, PTX > 10 nM shows about threefold of the cell growth inhibitory effect in gastric cancer cell than in human peritoneal mesothelial cells which mostly exists in peritoneal cavity [10]. Intravenous administration of 80 mg/m^2^ PTX reaches over 1.7 × 10^3^ nM in plasma [6]. Figure 3 in our study showed even 1 × 10^2^ nM PTX suppressed the cell growth inhibitory effect very well. 1.7 × 10^3^ nM PTX may be still more effective and unaffected by AGP or EM because of large free fraction of PTX. For this reason, negative effect of AGP and positive one of EM on cancer cells and normal cells may not be seen in whole body through peripheral circulation. In the previous report, when ovarian cancer patients with various AGP concentrations were treated with intravenous PTX modulating its pharmacokinetics by clindamycin binding to AGP, their toxicity was not increased [37].

The mechanism underlying the elevation in AGP concentrations observed in gastric cancer patients with PC remains unclear. AGP is synthesized mainly by hepatocytes in response to inflammation. Gastric cancer patients with PC are in a state of chronic inflammation, which may increase AGP levels. AGP concentrations have also been reported high in patients with idiopathic pulmonary fibrosis, but there were no correlations between AGP and C-reactive protein concentrations [17]. Elevations in AGP may also be due to a non-inflammatory mechanism. Extrahepatic synthesis of AGP has been reported [38], and AGP has been detected in malignant human tissue including stomach tumors [39]. Gastric cancer cells may produce AGP, and co-administration of PTX and EM may be effective in such a high-AGP environment.

## Conclusions

Co-administration of PTX and EM, which competes to bind AGP, might be an encouraging treatment for patients with metastatic advanced gastric cancer. Clinical trials are being planned to evaluate the effectiveness of EM supplementation in enhancing the effects of weekly PTX therapy for gastric cancer patients with PC.

## Abbreviations

PTX: Paclitaxel
PC: Peritoneal carcinomatosis
AGP: α1-Acid glycoprotein
EM: Erythromycin
5-FU: 5-Fluorouracil
MST: Median survival time
IP-PTX: Intraperitoneal PTX
TGF-β: Transforming growth factor-β
DMEM: Dulbecco’s modified Eagle’s medium
FBS: Fetal bovine serum
RPMI: Roswell Park Memorial Institute
MTT: 3-(4,5-Dimethylthiazol-2-yl)-2,5-diphenyltetrazolium bromide
SD: Standard deviation
IAP: Immunosuppressive protein
MRP: Multidrug resistance-associated protein
